# The Effects of Plastic Film Mulching on Maize Growth and Water Use in Dry and Rainy Years in Northeast China

**DOI:** 10.1371/journal.pone.0125781

**Published:** 2015-05-13

**Authors:** Jie Xu, Congfeng Li, Huitao Liu, Peilu Zhou, Zhiqiang Tao, Pu Wang, Qingfeng Meng, Ming Zhao

**Affiliations:** 1 Institute of Crop Science, Chinese Academy of Agricultural Sciences, Beijing, China; 2 Institute of Agricultural Environment and Research Center, Jilin Academy of Agricultural Sciences, Changchun, China; 3 College of Agronomy and Biotechnology, China Agricultural University, Beijing, China; Northwest A&F University, CHINA

## Abstract

Plastic film mulching (PM) has been widely used to improve maize (*Zea mays* L.) yields and water use efficiency (WUE) in Northeast China, but the effects of PM in a changing climate characterized by highly variable precipitation are not well understood. Six site-year field experiments were conducted in the dry and rainy years to investigate the effects of PM on maize growth, grain yield, and WUE in Northeast China. Compared to crops grown without PM treatment (control, CK), PM significantly increased the grain yield by 15-26% in the dry years, but no significant yield increase was observed in the rainy years. Yield increase in the dry years was mainly due to a large increase in dry matter accumulation pre-silking compared to the CK, which resulted from a greater dry matter accumulation rate due to the higher topsoil temperature and water content. As a result, the WUE of the crops that underwent PM (3.27 kg m^-3^) treatment was also increased by around 16% compared to the CK, although the overall evapotranspiration was similar between the two treatments. In the rainy years, due to frequent precipitation and scant sunshine, the topsoil temperature and water content in the field that received PM treatment was improved only at some stages and failed to cause higher dry matter accumulation, except at the 8^th^ leaf stage. Consequently, the grain yield and WUE were not improved by PM in the rainy years. In addition, we found that PM caused leaf senescence at the late growth stage in both dry and rainy years. Therefore, in practice, PM should be applied cautiously, especially when in-season precipitation is taken into account.

## Introduction

Northeast China is one of the most important maize-producing areas in China, accounting for 31% of the national maize area and 34% of the total amount of maize produced nationally [1]. Spring maize is one of the most popular grain crops cultivated by local farmers in this region [2]. As an important farming technique, plastic film mulching (PM) in spring maize production has been widely adopted in the region due to the significant benefits it confers in terms of yield increase and water conservation [3,4]. While the climate has varied greatly in recent years in the region, with substantial variability in annual precipitation [5–7], the effects of PM on maize production under different precipitation conditions remains poorly understood. Thus, insight into the effectiveness of PM under variable precipitation conditions could help farmers make informed decisions regarding its utility, and could improve maize production in the region.

Because Northeast China is located in the North high latitudes, low temperature after sowing often restrict maize emergence in practice [8]. Many studies have shown that PM is an effective strategy for promoting crop emergence because it can modify the soil microclimate by increasing the soil temperature [9–14]. Elevated soil temperatures can quicken crop emergence and growth to achieve the desired population structure at an earlier growth stage [15–17]; this can in turn maximize the absorption of solar radiation and enhance the yield [18,19]. However, other studies have shown that under some conditions PM throughout the growing season is detrimental to crop yield. For example, PM application for wheat was shown to accelerate leaf senescence late in the growing season, decreasing the 1,000-kernel weight [20]. In potato production, negative effects of full-season PM were observed at high air temperatures but not when the air temperature was low [21]. Because air temperature is closely related to precipitation, understanding the impact and underlying mechanisms of full-season PM on crop growth development, soil temperature, and grain yield under different precipitation conditions will be helpful for improving crop management in accordance with in-season precipitation.

Furthermore, another reason for adopting PM in Northeast China and similar areas is that it can retain precipitation, reduce water loss, and increase the water use efficiency (WUE). For example, the WUE of maize grown with PM was significantly increased by 23–25% on loessial tableland [22]. Further, in a semi-arid region of northern China, the WUE of PM-treated maize was 23–90% higher than that of unmulched maize [14]. In this case, PM increased the amount of soil-available water by restricting evaporation and elevating deep water by capillarity and vapor transfer to the layer usable for roots under arid and semi-arid conditions [23,24]. However, other studies have shown that PM can prevent precipitation infiltration during the middle and later growth stages of maize in the rainy season, thus reducing rainwater savings and working against the WUE [25,26]. It is predicted that variability in precipitation will continue to increase due to climate change [27], and there is little documentation on how soil water content and WUE might be affected by PM in poor versus rich precipitation years.

In this study, we analyzed the yield-PM relationship in Northeast China during dry and rainy years. The objectives of this study were to: (1) evaluate grain yield performance in plants treated with and without PM during dry and rainy years, (2) investigate changes in maize growth and development, and in soil temperature caused by PM during contrasted years, and (3) understand the changes in soil water content and WUE caused by PM during dry and rainy years. We have conducted six site-year field experiments in different precipitation contexts in Northeast China. For all studies, grain yield and yield components were investigated. The process data such as growth development, dry matter, leaf area index, and soil temperature and moisture was selected at Site 1 (Qianguo experimental station) in 2010 to represent the dry year. The process data from Site 4 (Gongzhuling experimental station) in 2013, which was close to Site 1 and exhibited similar ecological conditions, was selected to represent the rainy year. Comparing these different experiments may enable us to improve the use of PM and provide a theoretical basis and technical support for maize production in Northeast China and similar areas throughout the world.

## Materials and Methods

### Site Description

For all experimental sites, no specific permissions were required for these locations. The field studies did not involve endangered or protected species. Six field experiments at five sites were conducted in Qianguo (Site 1), Qianan (Site 2), Tongyu (Site 3), Gongzhuling (Site 4) and Nongan (Site 5) in Jilin Province, China. The preceding crops at all sites were all maize. Experiment year, location latitude and longitude, soil texture and selected chemical properties are shown in S1 Table.

At Site 1 in 2010, Site 2 in 2011, Site 3 in 2013 and Site 5 in 2014, the annual precipitation during maize growing season averaged 299 mm with a range from 278 to 328 mm, 9–24% less than the long-term (1960–2009) average (Table 1). These four site-year experiments were classified as dry years. At Site 2 in 2013 and Site 4 in 2013, the precipitation during maize growing season was 431 mm and 544 mm, 20% and 9% higher than the long-term average, respectively. These two site-year experiments were classified as rainy years. For all sites, the annual air temperature averaged 20.3°C during maize growing season.

**Table 1:** Monthly average precipitation and temperature during the maize growing season for all sites and the long-term (1960–2009) average.

Weather date	Site	Location	Year	Month					
				May	June	July	August	September	Total
Precipitation (mm)	Site1	Qianguo	Long-term (1960–2009)^a^	33.7	77.6	132.6	85.8	39.3	369
			2010 (Dry)	119	6.9	87.4	73.2	6.1	292.6
	Site 2	Qianan	Long-term (1960–2009)	27.9	71.5	135.7	90.7	33.7	359.5
			2011(Dry)	88.9	21	101.9	90.2	25.9	327.9
			2013 (Rainy)	33.7	69.2	158.7	148.7	20.6	430.9
	Site 3	Tongyu	Long-term (1960–2009)	28.3	71.4	127.2	82.3	28.4	337.6
			2013 (Dry)	54.9	47.1	64.9	90.2	20.8	277.9
	Site 4	Gongzhuling	Long-term (1960–2009)	52.5	95.2	168.2	139.7	51.4	507.1
			2013 (Rainy)	22.9	80.9	137.1	273.2	39.8	553.9
	Site 5	Nongan	Long-term (1960–2009)	62.6	93.3	127	82.3	22.6	387.7
			2014 (Dry)	92.7	73.6	52.2	43.9	34.1	296.5
Temperature (°C)	Site 1	Qianguo	Long-term (1960–2009)	15.6	21.1	23.8	22.1	15.6	19.6
			2010	16.3	25	23.3	22	17.1	20.7
	Site 2	Qianan	Long-term (1960–2009)	15.8	21.3	23.7	22	15.6	19.7
			2011	15.3	22.4	24.4	23.4	15.3	20.2
			2013	18.4	22.2	24.3	23	16.4	20.8
	Site 3	Tongyu	Long-term (1960–2009)	16.1	21.4	24	22.2	15.8	19.9
			2013	17.7	20.7	24.1	22.4	15.2	20
	Site 4	Gongzhuling	Long-term (1960–2009)	16.3	21.1	23.7	22.4	16.2	19.9
			2013	18.4	21.7	24	23	17	20.8
	Site 5	Nongan	Long-term (1960–2009)	16.3	21.4	23	21.7	15	19.5
			2014	14.8	22.7	22.7	21.6	14.3	19.2

^a^ The long-term value is the average precipitation or temperature from 1960 to 2009.

### Experimental Design and Crop Management

For the six field experiments, we applied two treatment conditions: no PM (control, CK) and PM. All treatments involved alternating wide and narrow rows of 80 cm and 40 cm at Site 1, 3, 4 and 5, 90 cm and 40 cm at Site 2. For PM treatment, the plastic film was laid out over the narrow rows. The CK treatment was non-mulched field.

All of the experiments consisted of a randomized complete block design with three replicates. The plot area varied from 30 m^2^ at Site 2 to 68 m^2^ at Site 5. The details of treatments including varieties and N, P, and K fertilizer application were shown in S2 Table. In each plot, the maize was planted 5 cm deep at a density of 45,000 at Site 3 to 75,000 plants ha^-1^ at Site 2. After sowing, the plastic film was laid out by hand. The mulch was drilled immediately as soon as the emergence of seeding, and was compacted around the seeding by soil to prevent ventilation four days later. Because of drought, both the PM and CK fields were irrigated two to three times with 65–100 mm of total amount using the microspray irrigation technique in dry years. No obvious nutrient, weed, pest, or disease stress was observed during the maize growing seasons at all sites.

### Sampling and Laboratory Procedures

To identify the stage of crop development, a standardized maize developmental staging system was used [28], and the date was recorded when more than 50% of the maize plants in each plot reached the following vegetative (VS) and reproductive (RS) stages: sowing time (ST), emergence stage (VE), the eighth leaf stage (V8), the twelfth leaf stage (V12), silking stage (R1), milking stage (R3), and physiological maturity (R6).

At Site 1 and 4, to measure the dynamics of dry matter accumulation, three adjacent plants (at least 1 m from the plot edge and 0.5 m from previous sampling sites) in each row were sampled randomly from each plot at V8, V12, R1, R3, and R6. The sampled plants were heated at 105°C for 30 minutes and dried at 75°C to a constant weight before weighing. The dry matter accumulation complied with a logistic equation, and the daily dry matter accumulation rate was calculated using the following Logistic Formula [29]:

$$\frac{dy}{dx}=\frac{abce^{-cx}}{{\left(1+be^{-cx}\right)}^{2}}$$ (1)

where *a* is the ultimate dry matter, *b* is the initial value, and *c* is the parameter for growth rate.

The leaf area index (LAI) of the sampled plants was measured immediately after harvest. For each leaf, the length and maximum width were measured, and the leaf area was estimated using the following equation [30]:

leafarea=length×width×0.75 (2)

The LAI was calculated using the following equation:

$$LAI=leaf\;area\;(m^{2}\;plant^{-1})\times plant\;density\;(plant\;ha^{-1})/10,000\;(m^{2}\;ha^{-1})$$ (3)

At Site 1 and 4, the soil water content was measured gravimetrically at 20-cm intervals at 0–100 cm from the soil surface in each plot at the ST, V8, V12, R1, and R6 stages. The soil moisture content at a depth of 0–100 cm from the surface in each plot was then calculated as the total soil water storage.

Seasonal evapotranspiration (ET) was determined using the formula [31,32]:

ET=ΔW+P+I (4)

Where Δ*W* is the change in soil water storage between sowing and harvesting, *P* is the precipitation during the crop growing season, and *I* is the total irrigation water quota. Surface runoff and deep drainage are neglected in most studies [33].

WUE was calculated as the grain yield using the following formula [34]:

$$WUE=\frac{Y}{ET}$$ (5)

where *Y* is the grain yield and *ET* is the total ET over the growing season.

For soil temperature measurements at Site 1 and 4, a set of mercury-in-glass geothermoments with bent stems (Hongxing Thermal Instruments, Hebei, China) were placed between the plant rows at soil depths of 5cm and 10 cm. The soil temperature was recorded at 8:00, 14:00, and 18:00 daily at the ST, VE, V8, V12, R1, and R6 growth stages. The mean daily soil temperature was calculated as the average of the three daily readings.

At physiological maturity for all sites, 7.2 m^2^ to 10 m^2^ of the crop area was harvested by hand from the four center rows in each plot. The ear number was counted in at least two rows with 6 m at harvest to determine the number of ears per ha. The 1,000-kernel weight was calculated as the average of three random samples of 500 kernels. The ear kernel number was recorded as the mean kernel number of ten ears from each replication. Grain yield was adjusted to 14% moisture.

### Data Analysis

For all sites, the mean grain yield and yield components between the CK and PM treatments were subjected to a one-way analysis of variance using SAS. Meanwhile, at Site 1 and 4, dry matter accumulation and LAI at each stage, and ET and WUE between the CK and PM treatments were also compared with the one-way analysis of variance.

## Results

### Grain Yield and Yield Components

The use of PM affected the maize yield differently in the dry and rainy years (Table 2). In the dry years, the grain yield under PM treatment averaged 10.5 Mg ha^-1^ (9.1–12.4 Mg ha^-1^), which was 19% higher than that under CK treatment with an average of 8.9 Mg ha^-1^ (7.4–10.7 Mg ha^-1^). However, in the rainy years, the grain yield under PM treatment ranged from 11.8 Mg ha^-1^ to12.7 Mg ha^-1^, with no significant difference compared to the CK treatment (10.9 Mg ha^-1^-12.3 Mg ha^-1^). The increased grain yield with PM in the dry years was mainly the result of an improved number of kernels per ear, which averaged 561.7 ear^-1^ (511.8–598.2 ear^-1^), or 12% higher than that for the CK treatment (465.3–523.1 ear^-1^), while no significant differences in the ears ha^-1^ were observed between the two treatments. At Site 5, the 1,000-grain weight in PM treatment was also 12% higher than the CK treatment. In the rainy years, all yield components were similar between the CK and PM-treated fields.

**Table 2:** Grain yield, and yield components of maize in the control (without plastic film mulching [PM] treatment; CK) and PM-treated crops at all experimental sites.

Year	Site	Location	Year	Treatment	Yield (Mg ha^-1^)	Ear number (plant ha^-1^)	Kernel number (ear^-1^)	1000-grain weight (g)
Dry years	Site 1	Qianguo	2010	CK	10.7b^a^	67545a	507.9b	355.9a
				PM	12.4a	67050a	589.6a	357.9a
	Site 2	Qianan	2011	CK	9.8b	63796a	509.4b	319.7a
				PM	11.3a	67479a	547.2a	321.3a
	Site 3	Tongyu	2013	CK	7.5b	43667a	523.1b	344.0a
				PM	9.1a	45000a	598.2a	360.6a
	Site 5	Nongan	2014	CK	7.4b	60667a	465.3b	270.7b
				PM	9.3a	63333a	511.8a	303.3a
Rainy years	Site 2	Qianan	2013	CK	10.9a	75000a	412.4a	371.3a
				PM	11.8a	75500a	437.6a	370.0a
	Site 4	Gongzhuling	2013	CK	12.3a	68085a	567.2a	333.5a
				PM	12.7a	69480a	556.7a	341.4a

^a^ Within two treatments in the same year means followed by the same letter are not significantly different at *P*<0.05.

### Dry Matter, Dry Matter Accumulation Rate, and LAI

**Fig 1:**
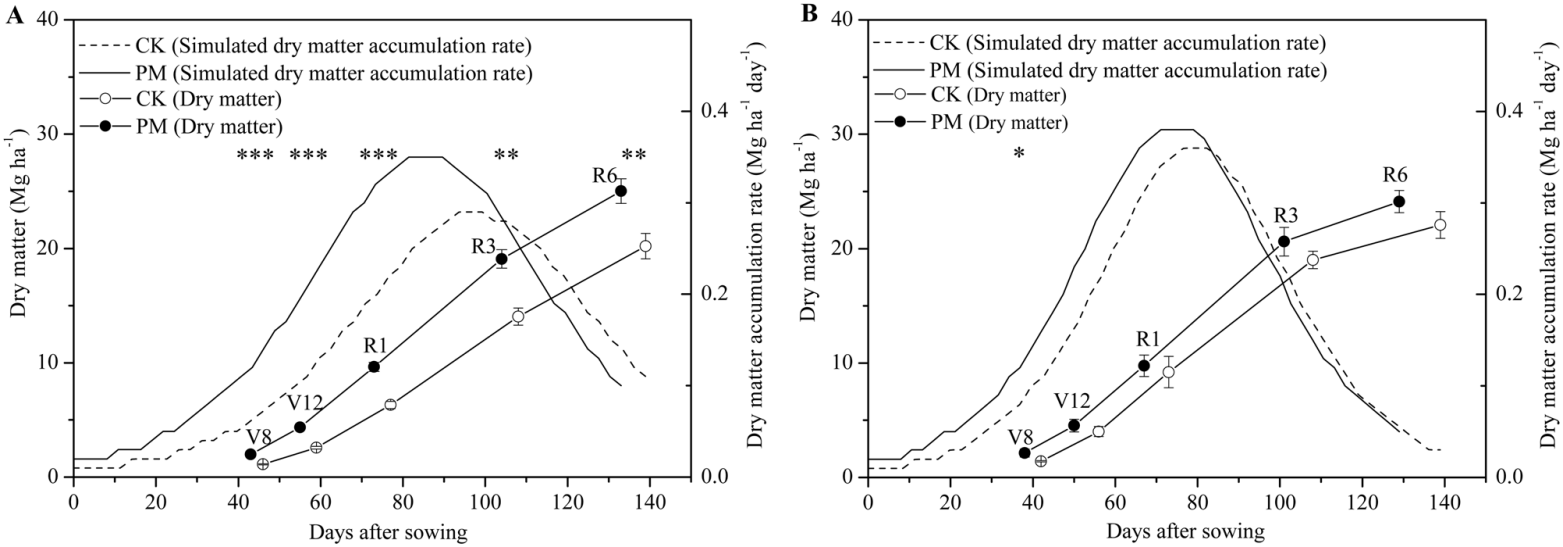
Dry matter accumulation at each growth stage and the simulated daily dry matter accumulation in the control (without plastic film mulching [PM] treatment; CK) and PM-treated crops in a dry year (Site 1 in 2010, A) and a rainy year (Site 4 in 2013, B). Vertical bars represent standard deviations of the means. *, significant at *P*<0.05; **, significant at *P*<0.01, *** significant at *P*<0.001. V8, the 8th leaf stage; V12, the 12th leaf stage; R1, silking stage; R3, milking stage; R6, physiological maturity.

**Fig 2:**
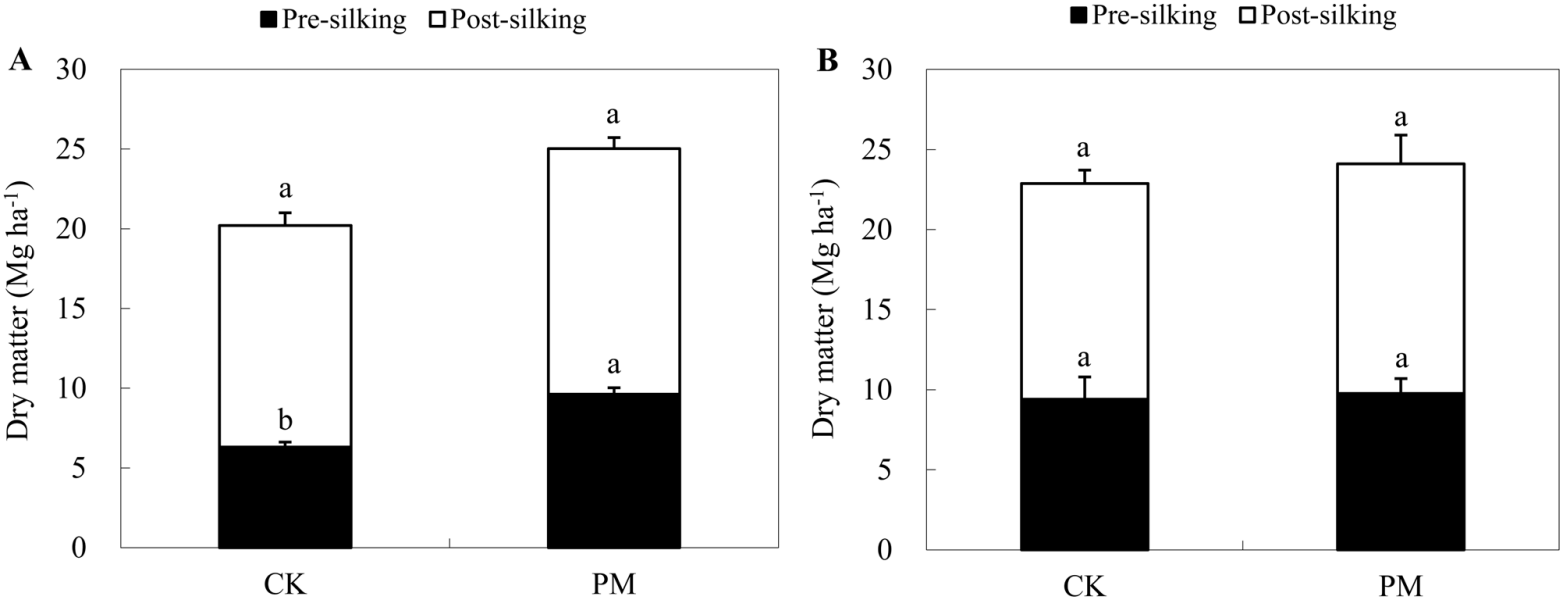
Dry matter accumulation pre- and post-silking in the control (without plastic film mulching [PM] treatment; CK) and PM-treated crops in a dry year (Site 1 in 2010, A) and a rainy year (Site 4 in 2013, B). Vertical bars represent standard deviations of the means. Different letters on the graph indicate significant differences at *P*<0.05.

The effects of PM on dry matter accumulation varied at different growth stages in years with different precipitation (Fig 1). In the dry year at Site 1, the amount of dry matter was significantly higher under PM conditions compared to CK conditions at each growth stage (V8, V12, R1, R3, and R6). The dry matter value at the V8, V12, R1, and R3 stages was 2.0 Mg ha^-1^, 4.4 Mg ha^-1^, 9.6 Mg ha^-1^, and 19.1 Mg ha^-1^, or 77%, 70%, 53%, and 36% higher than the CK values, respectively. At harvest, the total dry matter in the PM-treated field was 25.0 Mg ha^-1^, or 24% higher than that in the CK field (20.2 Mg ha^-1^). Furthermore, the dry matter accumulation pre-silking in the PM-treated field was significantly higher than that in the CK case, while no significant difference in dry matter accumulation was observed post-silking (Fig 2A). In contrast, in the rainy year at Site 4, PM only increased the dry matter significantly at the V8 stage; no significant improvement in dry matter was observed at the other stages. At the V8 stage, the amount of dry matter averaged 2.1 Mg ha^-1^, which is roughly 50% higher than the 1.4 Mg ha^-1^ recorded under CK conditions (Fig 1B). Furthermore, no significant dry matter accumulation was observed at either the pre- or post-silking stage in the rainy year (Fig 2B).

During pre-silking, dry matter accumulated fast in the PM-treated field compared to the CK field at both Site 1 and 4 in both years (Fig 1). At Site 1 in the dry year, the dry matter accumulation rate in the PM-treated field averaged 0.1 Mg ha^-1^ day^-1^ from sowing to the V8 stage, which was 88% higher than that for the CK field (Fig 1A). From V8 to R1, the dry matter accumulation rate in the PM-treated field was 0.2 Mg ha^-1^ day^-1^, which was 59% higher than that for the CK field. At Site 4 in the rainy year, the dry matter accumulation rate in the PM-treated field was 41% higher than that for the CK field from sowing to the V8 stage. However, it was only 11% higher in the PM-treated field compared to the CK field from V8 to R1 (Fig 1B). Post-silking, the dry matter accumulation rate in the PM field was high during the earlier stages, but decreased faster at Site 1. At Site 4, similar results for the change in dry matter accumulation rate were observed after silking.

**Fig 3:**
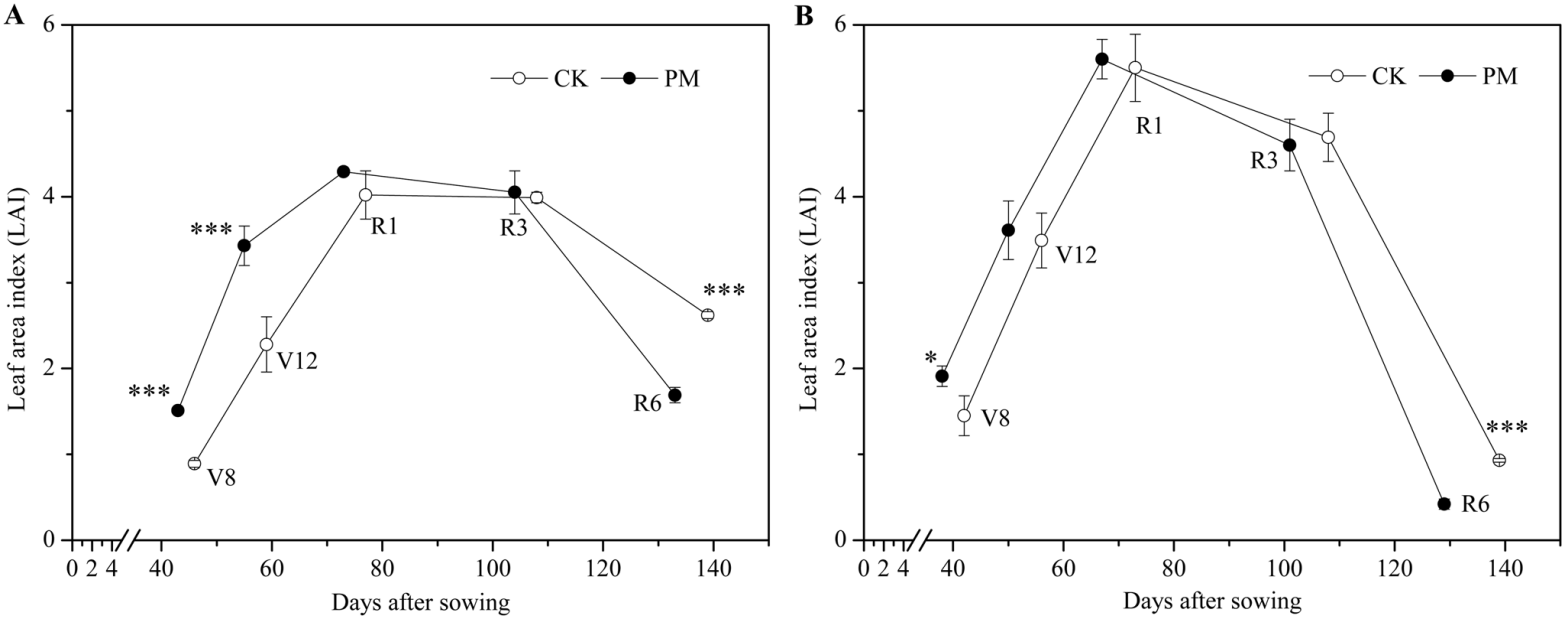
Leaf area index dynamics during the maize growth season in the control (without plastic film mulching [PM] treatment; CK) and PM-treated crops in a dry year (Site 1 in 2010, A) and a rainy year (Site 4 in 2013, B). Vertical bars represent standard deviations of the means. *, significant at *P*<0.05; **, significant at *P*<0.01; ***, significant at *P*<0.001. V8, the 8th leaf stage; V12, the 12th leaf stage; R1, silking stage; R3, milking stage; R6, physiological maturity.

Similar to the dry matter accumulation rate, the pre-silking LAI in the PM-treated crops was significantly higher at Site 1 in the dry year compared to that in the CK crops (Fig 3A). The LAI in the PM-treated crops was 1.51 at V8 and 3.43 at V12; these values are 70% and 50% higher, respectively, compared to those for the CK crops. At R1, the LAI was similar between the PM- and CK-treated crops. At Site 4 in the rainy year, PM only increased the LAI prior to the V8 stage, showing a value of 1.91, which was 32% higher than the value for the CK crops (1.45; Fig 3B). Post-silking, the LAI decreased rapidly in both Site 1 and Site 4. At maturity at Site 1 in the dry year, the LAI of the CK crops averaged 2.62, which was 35% higher than that of the PM-treated crops (1.69). At Site 4 in the rainy year, the final LAI for the CK crops was 0.93, which was 121% higher than that for the PM-treated crops (0.42). These data show that the presence of PM late in the season accelerated leaf senescence and led to a decrease in LAI at maturity under both rain conditions.

### Soil Temperature and Crop Development

**Fig 4:**
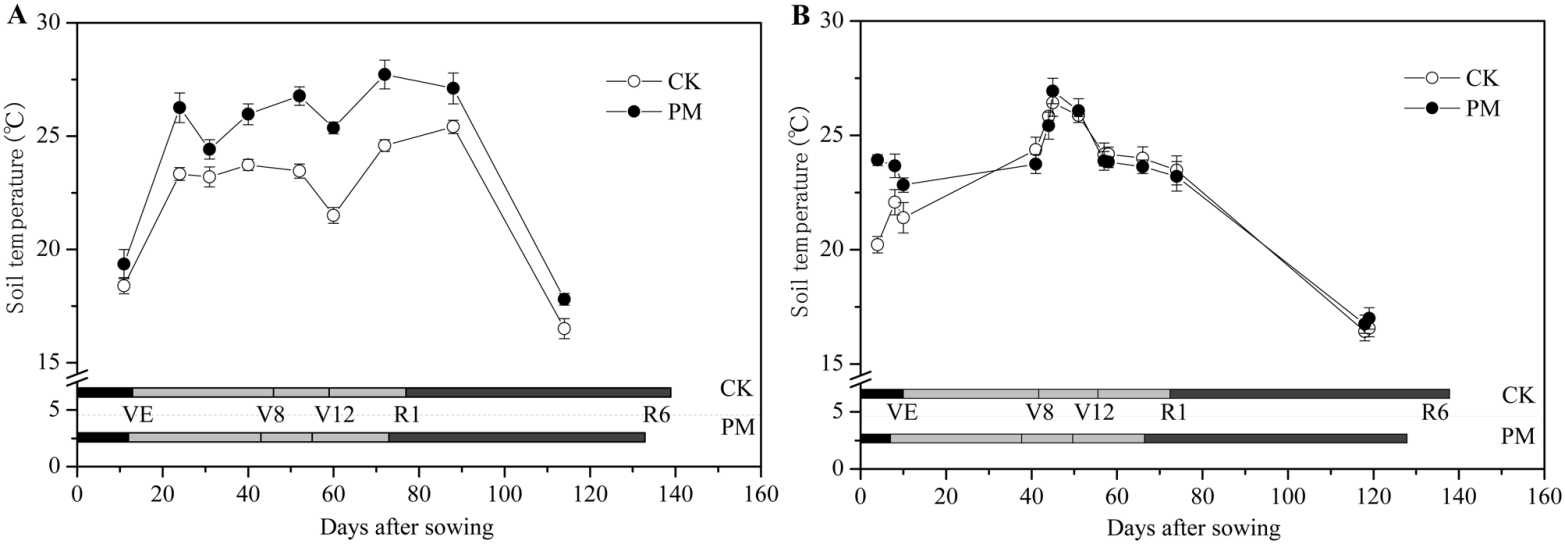
Soil temperature at a depth of 0–10 cm in the control (without plastic film mulching [PM] treatment; CK) and PM-treated crops in a dry year (Site 1 in 2010, A) and a rainy year (Site 4 in 2013, B). Vertical bars represent standard deviations of the means. VE, emergence stage; V8, the 8th leaf stage; V12, the 12th leaf stage; R1, silking stage; R3, milking stage; R6, physiological maturity.

Similar to dry matter accumulation, the effects of PM on the soil temperature varied during different growing stages under different precipitation (Fig 4). Due to the frequent precipitation from sowing to emergence (eight rainy days of a total of twelve days) at Site 1 in the dry year (S1 Fig), the soil temperature at a depth of 0–10 cm at Site 1 (18.4–19.4°C) was lower than that at Site 4 in the rainy year (20.2–23.9°C). Furthermore, PM increased the soil temperature by 3.7°C compared to the CK treatment at Site 4; this increase was significantly higher than the 1.0°C increase at Site 1. From emergence to V8, PM treatment increased the soil temperature by 2.2°C at Site 1 and 1.5°C at Site 4. From V8 to R1, PM elevated the soil temperature by 3.3°C at Site 1, whereas it did not affect the soil temperature significantly at Site 4. This was because precipitation occurred 30 times during the season from V8 to R1 at Site 4, while it only rained 18 times at Site 1. Post-silking, the soil temperature was 17.8°C for the PM-treated crops, which was an average of 1.3°C higher than that for the CK crops (16.5°C) at Site 1. At Site 4, the soil temperature was only 0.4°C higher for the PM-treated crops (16.9°C) compared to the CK crops (16.5°C) because of the frequent precipitation and scant sunshine (S1 Fig).

The increase in soil temperature caused by PM promoted significantly earlier seedling emergence and advancement to specific vegetative stages in both years (Fig 4). The duration of PM treatment from sowing to emergence was 12 days and 7 days at Site 1 and Site 4, advanced 1 day and 3 days compared to the corresponding CK treatment, respectively. Crops grown in the presence of PM required 31 days from emergence to V8 in both years, thus reducing the growth period by 3 days compared to the CK crops at Site 1 and 4 days at Site 4. From the V8 to the R1 stage at Site 1 and Site 4, the PM-treated plants required 30 days and 29 days, respectively, corresponding to a reduction of 1 day and 2 days compared to the CK treatment, respectively. Post-silking, PM shortened the grain-filling stage by 2 days to 4 days compared to CK treatment at Site 1 and Site 4, respectively.

### Soil Water Content, ET, and WUE

**Fig 5:**
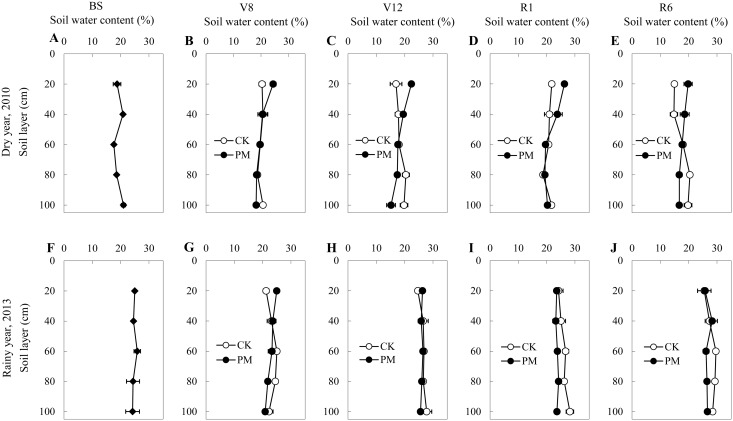
Soil water content at 0–100 cm from the surface in the control (without plastic film mulching [PM] treatment; CK) and PM-treated fields in a dry year (Site 1 in 2010) and a rainy year (Site 4 in 2013). BS, before sowing; V8, the 8th leaf stage; V12, the 12th leaf stage; R1, silking stage; R3, milking stage; R6, physiological maturity.

In both the dry (Site 1) and rainy (Site 4) years, PM treatment increased or maintained the soil water content for the top 0–20 cm of soil at each growth stage (Fig 5). In the dry year at Site 1, the soil water content at a depth of 0–20 cm was 24.37% at V8, 22.37% at V12, 26.27% at R1, and 19.80% at R6, reflecting an increase of 20%, 32%, 21%, and 32%, respectively, compared to CK treatment. Similarly, at a depth of 20–40 cm, soil moisture at the V12, R1, and R6 stages was 19.53%, 23.78%, and 18.60%, 10%, 13%, and 26% higher than in the CK crops, respectively, while it was similar between the two treatments at the V8 stage. The soil water content at a depth of 40–60 cm was similar between the PM and CK groups for each growth stage. With lower water supplementation from precipitation, the soil water content at a depth of 60–80 cm was 17.40% at V12 and 16.70% at harvest, which is 15% and 18% lower than that for the CK plots, respectively, while there was no significant difference between the two treatments at both V8 and R1 stages. At a depth of 80–100 cm in each growth stage, the soil moisture for the PM-treated plants was 12%, 23%, 6%, and 16% lower, respectively, compared to that for the CK plots. In the rainy year at Site 4, PM treatment significantly increased the soil water content in the top 0–20 cm of soil at the V8 stage only (24.98%), which is 18% higher than for CK treatment. When the rainy season started, there was no significant difference between the two treatments. At a depth of 20–40 cm, the soil moisture content was almost identical between the two treatments. At a depth of 40–60 cm, the soil moisture content was 23.32% at V8, 26.46% at V12, 23.79% at R1, and 26.13% at R6, or 7%, 1%, 11%, and 12% lower than that for the CK-treated plots, respectively. Similar trends were found at depths of 60–80 cm and 80–100 cm. These results show that the application of PM in a rainy year might block the infiltration of water from precipitation.

In the dry year at Site 1 and the rainy year at Site 4, the calculated ET was similar between the two treatments (Table 3). At Site 1 in the dry year, ET averaged around 380 mm, while it ranged from 506 mm to 526 mm at Site 4 in the rainy year. Accordingly, the calculated WUE of the PM-treated plants at Site 1 was 3.27 kg m^-3^, which represented an increase of 16% compared to the CK treatment (2.82 kg m^-3^), similar to the improvement in grain yield. At Site 4, the WUE of plants receiving both treatments averaged from 2.42 kg m^-3^ to 2.43 kg m^-3^, and no significant difference was observed between the two treatments.

**Table 3:** Evapotranspiration (ET) and water use efficiency (WUE) of the control (without plastic film mulching [PM] treatment; CK) and PM-treated crops in a dry year (Site 1 in 2010) and a rainy year (Site 4 in 2013).

Treatment	Site 1 (Qianguo)		Site 4 (Gongzhuling)	
	ET (mm)	WUE (kg m^-3^)	ET (mm)	WUE (kg m^-3^)
CK	379a^a^	2.82b	506a	2.43a
PM	380a	3.27a	526a	2.42a

^a^Within two treatments in the same year means followed by the same letter are not significantly different at *P*<0.05.

## Discussion

In this study, we found different effects of PM on maize yield and WUE according to the precipitation level in Northeast China (Tables 2 and 3). In the dry years, the maize yield following PM treatment was significant higher (19% more) than that following CK treatment. This yield increase as a result of PM is consistent with the results of other studies in arid and semi-arid areas. For example, compared to CK treatment, PM increased the maize yield by 23% in Mexico in 1988 [12], 17% in Bushland, TX, USA, in 1995 [35], 28% in 2010 and 88% in 2011 at the Changwu experimental station on the Loess Plateau of China [14], and 13% in the Weibei Highlands of China from 2008 to 2010 [36]. In contrast, in the rainy years, PM conferred no significant yield improvement (Table 2).

Our results at Site 1 indicate that the yield improvement in a dry year can be attributed to greater dry matter accumulation at an early stage of growth such as pre-silking (Fig 2). During this stage, the growth period was shortened (Fig 4). This suggests that the dry matter accumulation rate was improved substantially by PM (Fig 1). In this study, in accordance with the “greenhouse effect” [37], PM significantly increased the topsoil (0–10 cm) temperature and significantly accelerated maize growth and development. For example, PM promoted seedling emergence and each vegetative stage examined; it also improved the growth rate during the VS stage (Fig 1). This is because soil temperature controls the rate of maize development when the meristem is located underground until the meristem emerges from the soil surface at the jointing stage [38]. Meanwhile, the higher growth rate at earlier stage promoted a greater LAI [14] (Fig 3), which made it possible to maintain high levels of photosynthesis in the leaves [18]. This observation is consistent with those of other studies [14].

In our study, the water content in the top 20 cm of soil was higher in the PM than in the CK crops throughout the growth period in the dry year (Fig 5). In contrast, at a depth below 60 cm, the soil water content under CK conditions was higher at most growth stages. This was mainly because the plastic film kept the topsoil water content relatively stable by inhibiting water evaporation from the soil surface directly [17], and it enabled the movement of water from the deeper soil layers to the topsoil by capillary action and vapor transfer [13,39,40]. The augmented available soil water in the PM-treated field might have accelerated plant development, increasing the LAI and shoot biomass due to the stomata being more open than in the CK field [24]. Therefore, PM treatment was effective at mobilizing deep soil water when the precipitation level was low. In the dry year, the WUE of the PM-treated plants was 16% greater than that of the CK plants, although the ET level was similar between the two treatment conditions during the entire growth season (Table 3). This is mainly because of the 16% higher grain yield in the PM-treated plants compared to the CK plants. This increase in yield resulted from the development of more robust individual plants in the PM-treated field that were able to exploit deeper soil water under dry conditions [41]. This finding is in accordance with those of other studies, which showed that PM significantly increased the WUE of plants [14].

We also found that PM did not always result in significant improvements in maize yield, as observed during the rainy years (Table 2). In the rainy years, which was characterized by frequent precipitation and low photon flux density (PPFD), the topsoil temperature in the PM-treated field was similar to that in the CK field during certain important growth stages (e.g., from V8 to R1 and post-silking; Fig 4); therefore, maize growth with PM remained relatively consistent with the CK treatment. Furthermore, PM only increased the LAI and dry matter accumulation at the V8 stage (Figs 1 and 3). Once the rainy season began, no significant improvement in dry matter accumulation in the middle and later growth stages was observed for the PM-treated plants, and no obvious improvements in grain yield were observed.

During the rainy year at Site 4 in 2013, the topsoil water content was improved only at the early growth stage with PM (e.g., V8). When the rainy season started (e.g., continuous precipitation for four days, from day 96 to day 99 after sowing), the soil water content (40–100 cm) in the presence of PM was even lower than that under CK conditions (S1 Fig, Fig 5). This could be because the precipitation gathered on the flat surface of the plastic film and then evaporated directly [17], leaving the soil in the root zone drier than when no plastic was present. Meanwhile, the water on the surface of the plastic film would slow the rise of the soil temperature, and limit root development during the RS stage [22,42] while markedly decreasing the duration of the reproductive stage and green period. Thus, during a rainy year the PM should be removed temporarily to allow precipitation to fall onto the soil so that it can contribute to crop growth [14]. Because the PM prevented access to rainwater and soil temperature improvement, no increase in grain yield or WUE was observed in the rainy year. Finally, leaf senescence occurred rapidly in both dry and rainy years during the late growth season. Therefore, we recommend that PM should be removed from the field during the late growth stage in both dry and rainy years to both increase the grain yield and WUE of the crop plants.

Despite these observations, some aspects of the effects of PM on maize production should be further addressed. The effects of PM on maize production discussed in this study only based on one local variety, not fully incorporated other varieties. Previous studies have indicated that the PM effects would be magnified if the late-maturing varieties that had higher heat requirements were adopted [43]. The genotype x mulching interaction is therefore important to understand the underlying mechanism of PM effects such as growth duration or senescence onset. Meanwhile, the root x mulching interaction should also be taken into accounts in future. Recent studies have indicated that the maize root ideotype should include a large diameter primary root with few but long laterals, which could use available water in deeper soil strata over the growing season in most agricultural soils [44,45]. Grain yield therefore would be improved under terminal and intermittent drought. Furthermore, the ideotype of root structure architecture based on soil water profiles with PM observed in this study should also be taken into consideration. For example, we found soil water content in the top 20 cm soil was higher with PM in the dry year compared to the CK while it was lower at the depth below 60 cm at most growth stages (Fig 5). Finally, the economic benefits and environmental sustainability by PM need to be further analyzed. For example, PM would reduce the field management cost such as weeds control while it increases the material input.

## Conclusions

We found that PM induced different grain yields and WUE values between dry and rainy years. In the dry years, PM significantly improved the topsoil temperature and soil moisture, thereby promoting a higher LAI and rapid plant growth during the early VS stage. As a result, PM improved the accumulation of dry matter, leading to a significantly greater final biomass, grain yield, and WUE. In contrast, in the rainy years, PM improved these characteristics only in the early growth stage. In the middle and later growth stages, PM impacted the soil temperature and soil water content, preventing high biomass accumulation; thus, the final biomass, grain yield, and WUE values were similar to those for the CK treatment. In the dry years, PM treatment represented a more effective approach to improve both the WUE and maize yield in NEC, while PM did not improve maize production in the rainy years. Therefore, PM should used with caution, taking into account in-season precipitation.

## Supporting Information

S1 Fig Daily precipitation and mean temperature during the maize growing season in 2010 at site 1 (A) and 2013 site 4 (B). VE, emergence stage; V8, the 8th leaf stage; V12, the 12th leaf stage; R1, silking stage; R3, milking stage; R6, physiological maturity.
(TIF)

S1 Table Location, year, soil texture, and selected chemical properties in the top 20-cm soil layer at all sites in Northeast China. (DOCX)

S2 Table Variety and fertilizer rate at all sites in Northeast China. (DOC)
