# Supplementary figures and images for: The Effects of Plastic Film Mulching on Maize Growth and Water Use in Dry and Rainy Years in Northeast China
Source: PLoS One. 2015 May 13;10(5):e0125781. doi: 10.1371/journal.pone.0125781 (PMC4430173; doi:10.1371/journal.pone.0125781)

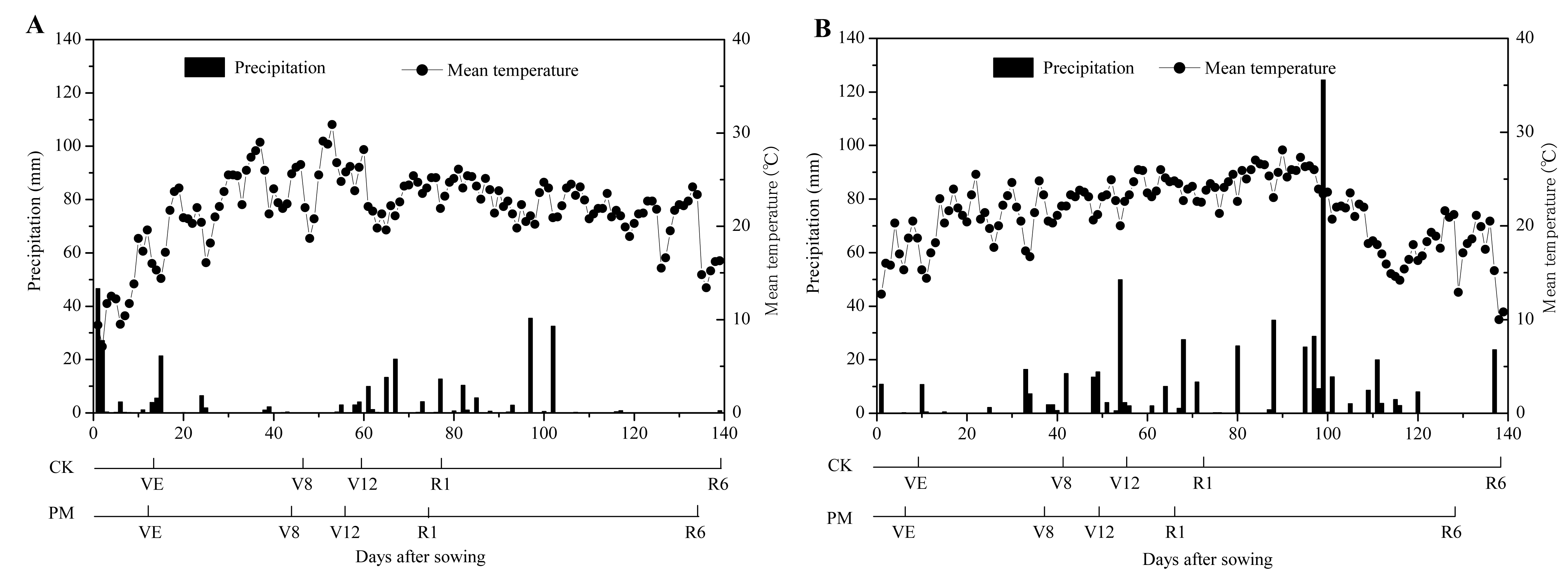

Supplement: S1 Fig — VE, emergence stage; V8, the 8th leaf stage; V12, the 12th leaf stage; R1, silking stage; R3, milking stage; R6, physiological maturity. (TIF) [file pone.0125781.s001.tif]
