# Supplementary material for: The Effects of Plastic Film Mulching on Maize Growth and Water Use in Dry and Rainy Years in Northeast China
Source: PLoS One. 2015 May 13;10(5):e0125781. doi: 10.1371/journal.pone.0125781 (PMC4430173; doi:10.1371/journal.pone.0125781)
Supplement: S1 Table — (DOCX) [file pone.0125781.s002.docx]

**S1 Table.** Location, year, soil texture, and selected chemical properties in the top 20-cm soil layer at all sites in Northeast China.

| **Sites** | **Location** | **Latitude and longitude** | **Year** | **Soil texture** | **Soil organic**  **matter**  **(g kg^-1^)** | **Total N**  **(g kg^-1^)** | **Available N**  **(mg kg^-1^)** | **Olsen-P**  **(mg kg^-1^)** | **NH_4_OAc-K**  **(mg kg^-1^)** |
| --- | --- | --- | --- | --- | --- | --- | --- | --- | --- |
| Site 1 | Qianguo | 44.29^o^N, 126.44^o^E | 2010 | Chernozem | 19.7 | 1.2 | 126.7 | 30.4 | 148.6 |
| Site 2 | Qianan | 43.29^o^N, 124.48^o^E | 2011, 2013 | Chernozem | 17.6 | 1.1 | 126.7 | 26.4 | 125.3 |
| Site 3 | Tongyu | 44.25^o^N, 123.52^o^E | 2013 | Chernozem | 16.6 | 1.2 | 105.5 | 39.6 | 158.7 |
| Site 4 | Gongzhuling | 43.29^o^N, 124.48^o^E | 2013 | Black soil | 26.2 | 1.3 | 114.2 | 34.5 | 150.5 |
| Site 5 | Nongan | 44.09^o^N, 125.11^o^E | 2014 | Chernozem | 20.7 | 1.3 | 120.6 | 45.8 | 136.5 |

.
