# Supplementary material for: The Effects of Plastic Film Mulching on Maize Growth and Water Use in Dry and Rainy Years in Northeast China
Source: PLoS One. 2015 May 13;10(5):e0125781. doi: 10.1371/journal.pone.0125781 (PMC4430173; doi:10.1371/journal.pone.0125781)
Supplement: S2 Table — (DOC) [file pone.0125781.s003.doc]

**S2 Table.** Variety and fertilizer rate at all sites in Northeast China.

| **Sites** | **Location** | **Year** | **Variety** | **Urea**  **(**kg N ha^-1^**)** | **Phosphorus**  **(**kg P_2_O_5_ ha^-1^**)** | **Potassium**  **(**kg K_2_O ha^-1^**)** |
| --- | --- | --- | --- | --- | --- | --- |
| Site 1 | Qiaguo | 2010 | Xianyu335 | 225 (40% base : 30% V8: 30% R1) | 100 | 110 |
| Site 2 | Qianan  Qianan | 2011 | Nonghua101 | 225 (33% base : 33% V8: 34% R1) | 100 | 110 |
|  |  | 2013 | Nonghua101 | 225 (33% base : 33% V8: 34% R1) | 100 | 110 |
| Site 3 | Tongyu | 2013 | Xianyu335 | 165 (40% base : 30% V6: 30% R1) | 90 | 100 |
| Site 4 | Gongzhuling | 2013 | Zhongdan909 | 250 (40% base : 30% V8: 30% R1) | 100 | 120 |
| Site 5 | Nongan | 2014 | Xianyu335 | 165 (40% base : 30% V6: 30% R1) | 80 | 100 |
